# Skin epidermal and microvascular properties at the sacrum and heel assessed with optical coherence tomography

**DOI:** 10.1159/000550488

**Published:** 2026-01-19

**Authors:** Ralph J. F. H. Gordon, Charlotte E. Stevens, Peter R. Worsley, Davide Filingeri

**Affiliations:** a ThermosenseLab, Skin Sensing Research Group, School of Health Sciences, University of Southampton, Southampton, UK; b PressureLab, Skin Sensing Research Group, School of Health Sciences, University of Southampton, Southampton, UK

**Keywords:** Pressure ulcer, tissue viability, microvasculature, skin aging, epidermal properties

## Abstract

**Introduction:**

The sacrum and posterior heel are two common areas where skin is exposed to pressure and shear forces which could lead to damage in the form of pressure ulcers. Despite their vulnerability, relatively few studies have explored their structural and physiological properties which predispose them to damage. The aim of this study was to characterise age- and anatomical site differences in the skin epidermal properties and microvasculature using optical coherence tomography (OCT), in younger and older adults.

**Methods:**

Twenty-two younger (18-35 years) and 19 older healthy adults (55-70 years) completed three experimental visits, comprising of non-invasive OCT imaging to characterize skin epidermal properties and microvascular density. Extracted parameters from the captured images were optical attenuation coefficient (OAC), dermal brightness, epidermal thickness, surface roughness (Rq), microvascular plexus depth (µm) and vascular density (%).

**Results:**

There were significant differences in the epidermal structural properties between the sacrum and heel across all parameters (P<0.001), except for roughness (P>0.05). The vascular density profiles plotted against skin depth were also significantly different between anatomical sites (P<0.001), with peak vascular density being more superficial in the heel: ˜0.2mm vs. sacrum: ˜0.4mm; (P<0.001). The sacrum had a greater maximum vascular density (˜9%) compared to the heel (˜7%; P<0.001). No significant differences were observed between the age groups (P>0.05).

**Conclusion:**

Distinct differences were observed for epidermal and vascular density measurements between heel and sacral skin sites. However, there were no age-related differences, which may be associated with the older adult range (55-70 years) and healthy status. The structural and microvasculature insights could aid in the design of therapeutic interventions to promote tissue viability and health such as site-specific dressing and textiles.

## Introduction

Pressure ulcers (PUs) are defined as localized damage to the skin and/or underlying tissue, resulting from prolonged pressure or pressure in combination with shear [1], usually over a bony prominence or related to the attachment of medical or other devices [2, 3]. The financial burden associated with the treatment of PUs in the United Kingdom remains high [4, 5]. In addition, once established, these wounds result in debilitating outcomes and a worsening of the quality of life for patients and carers [6]. Accordingly, an improved understanding of the fundamental mechanisms underlying the physiological structure and function of human skin at sites vulnerable to mechanical loading and shear, could provide insight for personalised solutions to prevent these wounds and improve patient care and quality of life.

Several healthcare technologies have been developed to help to prevent, delay, or mitigate the severity of PU. These include pressure redistribution surfaces, such as mattresses, other support surfaces, and monitoring devices, that try to detect and offload pressure from skin sites at-risk of PU such as the sacrum and heel [7–9]. Alongside pressure redistribution and offloading, the management of the microclimate at the interface between the skin and the support surface has received increasing attention [3]. This is largely due to evidence indicating that an increase in skin temperature is associated with the degradation of soft tissues leading to potential deep tissue injury [10, 11]. Conversely, animal models indicate that reducing the temperature at the skin interface may reduce the magnitude of tissue damage [10, 11], thereby acting in a protective capacity to maintain tissue viability. This has informed the design of several support interfaces and surfaces delivering local cooling via microclimate management systems [12]. However, evidence underlying their efficacy across different populations remain limited. It is unclear whether interventions should be adjusted based on factors such as age and skin site. This knowledge gap is driven by a lack of normative data on age- and body site-dependent differences in skin epidermal properties and microvascular density, which could modulate the efficacy of localised cooling therapy for PU prevention.

Throughout the life course, the aging process alters the functionality of human anatomical and physiological systems, including the skin, the largest organ in the body [13]. The process of skin aging is distinguished by intrinsic or extrinsic factors. Intrinsic factors comprise a complex series of biological functions but are largely attributed to cellular senescence and genetic influences [14–16], whilst extrinsic factors are largely derived from external interactions with the environment e.g., from exposure to the sun and ultraviolet exposure [16–18]. The characteristics of skin aging are well documented, including the presence of wrinkles, atrophy, rougher texture, a loss of elasticity and changes to pigmentation [19, 20], in addition to a decrease in the quality of the epidermis [14]. Yet, knowledge of the direct contribution aged skin may have on the prevalence of PU development remains limited, despite aged skin being recognised and an important indirect contributor in PU prevalence [2, 21]. PUs commonly develop over bonny prominences [2], and it has been widely reported that nearly 30% of pressure ulcers occur at the heel, with this region being the second most likely area for pressure damage after the sacrum [8, 22, 23]. As opposed to the thick skin of the plantar heel, the posterior heal has thin striated skin, which is very susceptible to pressure- and shear-induced damage [24]. Histopathology remains the clinical standard for precisely characterising skin aging [25], however, anatomical site and age specific comparisons of the two most prevalent areas of PU development (scrum and the heel), remain limited.

Optical coherence tomography (OCT) is a non-invasive imaging technique using light interference, that allows high resolution cross-section imaging of biological structures *in vivo* [26–29]. It has received increasing attention in dermatology, cancer diagnoses [27], and imaging of active wound healing [30]. Conventional OCT can provide structural information on the skin tissue’s epidermis and dermis, specifically epidermal thickness, skin surface roughness (Rq) and the optical attenuation coefficient (OAC), which is thought to be linked to the type of collagen and its density [26]. One of the recent functional extensions of OCT is the ability to detect and visualise cutaneous blood vessels through motion detection of red blood cells. This is known as dynamic-OCT (D-OCT), which is based on the principles of speckle variance. Research has been conducted to map a wide range of distinct anatomical skin sites, for example, in young individuals (18-35 years) [31]. Maiti et al [31] showed that changes in body posture (overextending the skin when moving joints to full capacity) altered thickness, roughness and undulation patterns of the skin, with thicker skin observed in load bearing regions (e.g., foot) compared to non-load bearing skin (e.g., chest) [31]. However, there is limited normative data identifying the spatial and temporal changes in structural and dynamic parameters of the skin, specifically at the sacrum and posterior heel, using D-OCT.

The aim of this study was to characterise age- and anatomical site differences in skin epidermal properties and microvascular density using OCT, in younger and older adults. It was hypothesised that age and skin site would interact such that epidermal and microvascular properties would be distinct between the sacrum and the heel, with older adults expressing characteristic age-related changes in these properties, such as, reduced epidermal thickness and an attenuated perfusion of the microvasculature [32].

## Methods

### Overview

Participants attended the laboratory within the Clinical Academic Facility located at Southampton General Hospital (Southampton, UK), to undertake three experimental visits. Data were collected in triplicate to accommodate an evaluation of the test-re-test repeatability of the methodology. Each visit was separated by a minimum of 24 hours. During the visits, participant’s skin over the sacrum and posterior heel were non-invasively imaged using OCT to characterize skin epidermal properties and microvascular density. The ambient conditions of the laboratory were maintained at 21±1°C.

### Participants

Twenty-two younger (9F/13M; 24±4 years; 176±9 cm; 71±9 kg) and 19 older (11F/8M; 65±4; 171±10; 70±14) healthy adults (assessed via a health screening questionnaire and self-reporting) provided informed consent prior to taking part. Ethical approval was granted by the University of Southampton Ethics Committee (Ethics and Research Governance Online: 88984) and all practices and procedures were carried out in accordance with the latest iteration of the *Declaration of Helsinki*. These data form part of a wider investigation into the influence of localized cooling on the skin’s microvascular and structural responses to sustained mechanical loading and shearing, as such the sample size justification is based on the calculations of Gordon et al., [12], with a minimum N=18 target recruitment per group (power α = 0.05; β = 0.80). Participants were included in the study if they were aged between 18-35 years (younger group) and 55-70 years (older group), male or female and physically active performing exercise at least 1-3 times per week. Participants were excluded from taking part if they suffered from cardiovascular, metabolic, and neurological disorders and/or comorbidities, e.g., diabetes, hypertension, Raynaud’s disease, skin conditions (e.g., eczema) or they smoked/vaped.

### Measurements and skin parameters

Non-invasive OCT imaging was used to measure the skin’s epidermal and microvascular properties at two sites, i.e. the sacrum and heel, using a VivoSight® hand-held scanner (Michelson Diagnostics Ltd., Maidstone, Kent, UK), which utilises a near-infrared wavelength laser source (1305 nm with a Class 1M (EN 60825-1). The principle of OCT has been previously described [27], but it is based on low-coherence interferometry. The OCT system incorporates a dynamic mode (D-OCT) based on the principles of speckle variance OCT, which is capable of imaging the microvasculature in the superficial dermis [31, 33]. Infrared light is used to illuminate the tissue, with the tissue of interest reflecting light in a backscattering geometry, which is compared with light from a reference path, producing a signal in the form of an axial scan (A-scan). The A-scan provides information on the profile of the underlying tissue microstructure by describing the relative position and intensity of the reflecting structures.

A total of 120 images with 50 μm spacing were acquired as a 6 × 6 × 2 mm^3^ (width × length × depth) stack. All D-OCT scans were taken in duplicate, by the same investigator. Spacers at the probe interface were used to standardise and optimise the focal point of the epidermis during scanning, with minimal but consistent pressure applied to the probe during image capture.

Skin parameters were extracted from the captured images using the proprietary VivoTools software to determine the optical attenuation coefficient (OAC), dermal brightness, epidermal thickness, surface roughness (Rq), microvascular plexus depth (µm) and vascular density (%). The OAC measures the rate at which the OCT signal brightness falls with depth below the skin surface, which is likely due to scattering and absorption of the light source from the surrounding tissues. The OAC is therefore the slope of the light intensity vs. depth in the upper dermis, the steeper the slope the faster the light will be attenuated by the tissue. The OAC, dermal brightness, epidermal thickness and microvascular plexus depth are calculated from the A-scan and dependent upon the OCT detecting the surface of the skin, epidermis and the dermis [26, 33]. It should be noted; there is a variation in the N samples analysed for the skin parameters (see Table 1). This is likely due to either the surface of the skin or epidermis not being detected. Rq is an estimation of the root mean square variation of surface height. Vascular density provides a measure of the active vasculature in the skin as a function of depth below the skin surface, ranging from 0.0 (no vessels) to 1.0 (100% of the tissue is vessels). Vascular density as an index of blood flow is calculated by averaging the brightness of D-OCT image across all pixels in the en-face image, at the selected depth. It detects blood flow by measuring which OCT pixels are changing rapidly due to flow of blood. Therefore, vascular density is an estimate of the % of the measured tissue comprising blood vessels.

**Fig. 1:**
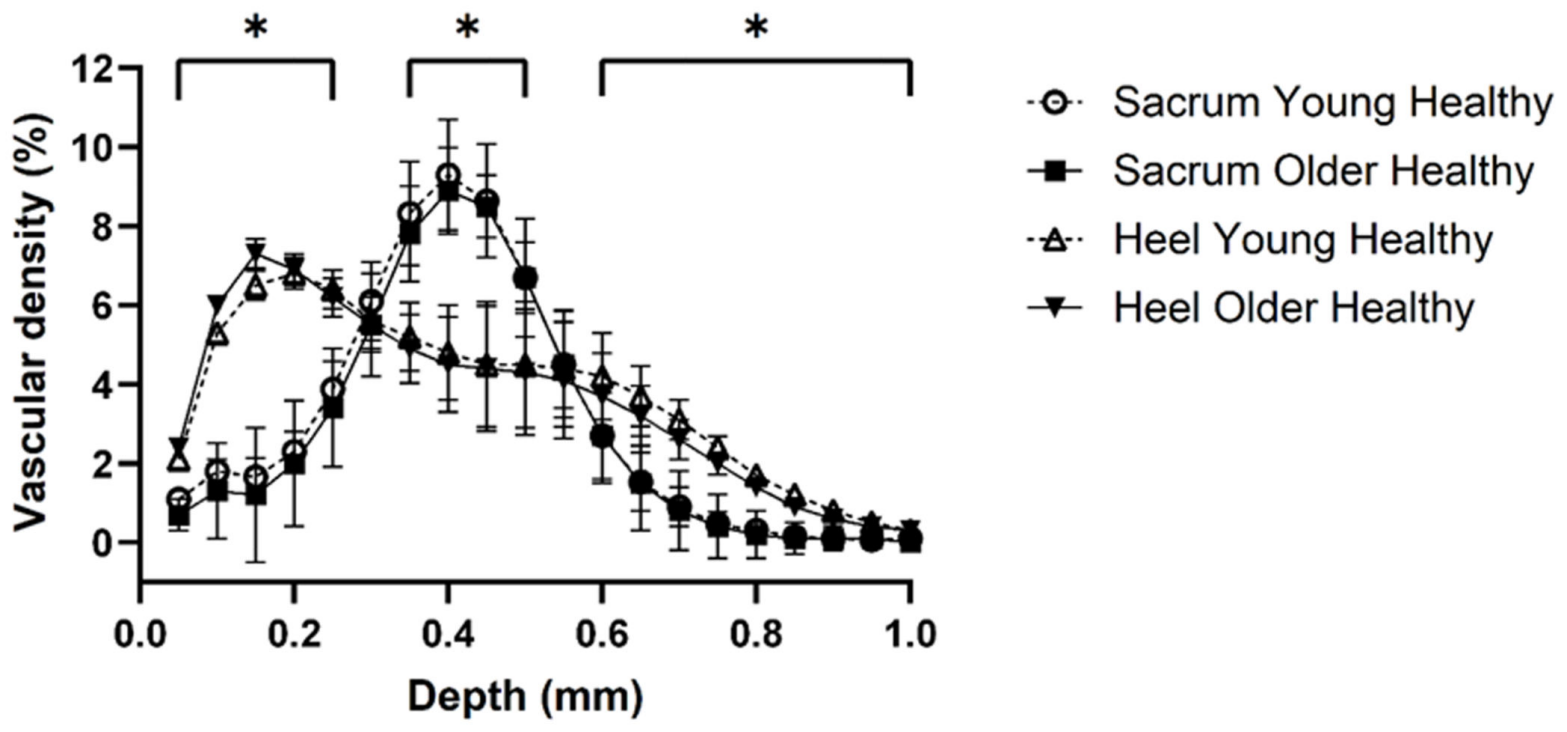
Vascular density profiles of the sacrum and the heel plotted against depth. Data are means±SD for *n* = 22 (young healthy) and *n* = 19 (older healthy). * Denotes statistically significant difference (P < 0.001) for *Bonferroni* corrected pairwise comparisons between anatomical sites (collapsed by age group).

All scans were taken in duplicate and averaged to obtain a mean value for that visit. The mean values for each of the three experimental visits were then averaged, for all measured variables (see Table 1 and Fig. 1). To establish the test-retest reliability of the methodology, the values from each experimental visit were used to calculate the intraclass correlation coefficient (ICC).

### Experimental Procedures

Participants were requested to attend the laboratory wearing loose, comfortable fitting clothing. Upon arrival, participants were seated whilst they adjusted (10 minutes) to the ambient conditions of the laboratory (21±1°C) before height and body mass were recorded (Model 874; Seca GmbH, Hamburg, Germany).

Following the pre-experimental checks, participants were seated on a hospital bed in a semi-recumbent position supported by pillows and a back rest. To standardise the position of the foot and ankle, an adjustable orthotic foot brace was strapped on to the right foot (all OCT measurements were taken from the right foot) and set to a neutral plantar grade position (90°).

To obtain OCT measurements from the skin over the heel, participants flexed their right leg to approximately 90°, abducted the thigh and rested the lower extremity of the leg (between the knee to the ankle) on the upper extremity of their left leg (between the hip and the knee). The imagining site was identified as the midpoint of the calcaneus and marked with non-permanent ink to allow consistent imaging for repeated measurements.

Once all scans were completed at the heel, participants repositioned themselves on the hospital bed to lie down in a prone position. Pillows were provided to support the pelvic and lumbar regions and to flatten the sacroiliac joint, reducing any pronounced lordosis. The sacrum was located via palpation and marked with non-permanent ink to allow consistent placement between measurements. Once the sacrum had been successfully located OCT measurements were taken.

### Statistical analysis

Descriptive data are reported as means and 95% confidence intervals (CI), unless otherwise stated. Data were assessed for normality of distribution using the Kolmogorov–Smirnov test.

Separate two-way mixed-model ANOVAs were used to evaluate the independent and interactive effects of age (younger vs. older) and anatomical site (sacrum vs. heel) on the dependant variables, namely OAC, dermal brightness, epidermal thickness, Rq and microvascular plexus depth.

Vascular density was analysed using a three-way mixed-model ANOVA to evaluate the independent and interactive effects of age (younger vs. older), anatomical site (sacrum vs. heel) and of microvasculature depth (20 data points starting at 0.05mm up to 1.0mm). Violations of sphericity were corrected for using the Greenhouse- Geiser adjustment when appropriate. Following a statistically significant F value, for the dependant variable vascular density, *post hoc* analyses were conducted to evaluate differences between anatomical sites at different microvascular depths using stepwise *Bonferroni*-corrected paired *t* test.

Finally, to assess test-rest reliability of the OCT measurements across the 3 separate experimental visits, ICC were calculated for all dependant variables. ICC estimates and their 95% CI were calculated based on a mean rating (*k* = 3), absolute agreement, two-way mixed effects model. The 95% CI estimates of the ICC were used to qualify poor (<0.5), moderate (0.5-0.75), good (0.7-0.9) and excellent reliability (>0.9). The 95% CI estimates of the ICC are considered to provide a more objective estimate of the ICC, rather than relying exclusively on the expected true value of the ICC [34].

The statistical significance level was set at P < 0.05. Statistical analysis was completed using IBM SPSS (version 30; IBM Corp., Armonk, N.Y., USA).

## Results

### Skin epidermal and microvascular properties

Table 1 shows skin epidermal properties and vascular density data. There was a main effect of anatomical site (sacrum vs. heel) on the OAC, dermal brightness, epidermal thickness and plexus depth (P < 0.001) but not on Rq (P > 0.05; Table 1). Specifically, the OAC, dermal brightness, and the plexus depth were greater in the skin of the sacrum compared to the heel, whilst epidermal thickness was greater in the heel compared to the sacrum. No main effects of age nor any interaction effects were observed on OAC, dermal brightness, epidermal thickness, plexus depth and Rq (P > 0.05; Table 1).

Figure 1 depicts the vascular density profiles plotted as a function of depth for the sacrum and heel, respectively, for both the younger (N=22) and older cohorts (N=19). There were main effects of anatomical site (sacrum vs heel; P < 0.001) and depth of vascular expression (P < 0.001), as well as an interaction effect for anatomical site x depth (P < 0.001). No main effect of age (younger healthy vs. older healthy; P = 0.422) nor interaction effects for age x depth (P = 0.794) or anatomical site x depth x age (P = 0.502), were observed. *Post hoc* pairwise comparisons (collapsed by age group) indicated statistically significant differences (P < 0.001) between the sacrum and the heel at all but two depths, 0.3 mm (P = 0.522) and 0.55 mm (P = 0.572; Fig. 1).

### Test-rest reliability of the OCT measurements

Table 2 shows the ICC estimates for OAC, dermal brightness epidermal thickness, Rq and plexus depth for the sacrum and heel. For brevity of analysis, ICC estimates were calculated and have been presented collapsed by age group. Test-retest reliability across the 5 parameters evaluated was generally “good”, with higher frequency of “good to excellent” scores for the heel (i.e. 4 out of 5 parameters) than the sacrum (2 out of 5 parameters; see Table 2).

Table 3 shows the ICC estimates for vascular density in the sacrum and the heel at different depths, collapsed by age group. Test-retest reliability across the 20 depths evaluated was generally “good”, with higher frequency of “good to excellent” scores for the heel (i.e. 9 out of 20 depths) than then sacrum (7 out of 20 depths; see Table 3).

## Discussion

This study aimed to assess age-dependent differences in skin epidermal and microvascular properties at the sacrum and heel assessed with optical coherence tomography. Leveraging a large cohort of younger (mean age: 24y) and older adults (mean age: 65y), and by collecting triplicate measurements of skin parameters on separate days to assess test-retest reliability, our findings indicated that skin epidermal and microvascular properties varied largely as a function of skin site, but not ageing up to ˜70y. Specifically, we found that in both younger and older adults, the heel had lower OAC, dermal brightness and Rq, and greater epidermal thickness, than the sacrum. We also found that, irrespective of age, the microvascular plexus of the sacrum and heel presented distinct density by depth profiles. Specifically, the heel presented a more superficial microvascular plexus (˜120 µm) than the sacrum (˜270µm), with its maximum vascular density peaking at shallower depths (heel: ˜0.2mm vs. sacrum: ˜0.4mm); although the sacrum presented a slightly greater maximum vascular density (˜9%) than the heel (˜7%, Fig. 1). Taken together, our findings partially support our initial hypothesis that skin epidermal and microvascular properties are distinct between the sacrum and the heel. In contrast, the data does not support our hypothesis that more aged skin significantly interacts with these epidermal and microvascular properties, according to skin site.

The characteristics of skin aging are well documented, with the mechanisms for skin aging being two-fold, the influence of extrinsic factors from the external environment e.g., ultraviolet radiation, and intrinsic factors such as cellular senescence [15] or oxidative stress [16]. In the present study, extrinsic factors are likely to have had a minimal impact, given the locations of the skin sites assessed (i.e., the skin of the sacrum and the posterior heel are rarely exposed to sunlight [17, 18]. Age related changes (20–40-year-olds vs. 60–80-year-olds) have been found in epidermal thickness *in vivo* using OCT [35] and an increase in dermal brightness [36]. We didn’t observe any statistical differences in epidermal thickness in either anatomical site; however, epidermal thickness in the heel was on average 44.8 μm thinner in the older healthy cohort compared to the younger healthy, as shown in Table 1. A further explanation for the lack of age-related differences may be in the participant cohort. We recruited healthy older individuals to an upper age limit of 70 years. Therefore, the skin of our older participants may not have been sufficiently vulnerable to exhibit age-related declines in functionality.

Due to technical difficulties extracting epidermal thickness, the data are for n=11 only, therefore, the lack of statistical difference may be due to an underpowered sample size rather than structural changes in the aged skin, which would support the literature that have assessed other anatomical sites such as the volar forearm [35, 37, 38], neck [17, 38], buttock [35, 38], upper arm [38], chest [17, 35, 38] and hand [38, 39]. It is worth highlighting, however, that despite the advances in the use of OCT for clinical applications [28] and efforts to map morphological characteristics of different anatomical skin sites using OCT [31], limited reference data exists for the skin over the sacrum and posterior heel. To the author’s knowledge, our data provides a novel characterisation using D-OCT, specifically at these anatomical sites. An absence of age-related changes may suggest that age is not a significant consideration for structural and morphological skin parameters when implementing therapeutic treatments in the prevention of pressure ulcers, up to 70 years old. Future research should investigate how these data compared to more aged skin (>70 years).

Vascular density profiles in the different anatomical sites appear to be distinct, as shown in Figure 1, where vascular density was shown to change as a function of depth. Not only does vascular density change as a function of depth, but there are distinct differences dependant on the skin site. For example, peak vascular density (˜9%) in the sacrum occurs at a depth of 0.4 mm, compared to peak vascular density (˜7%) in the heel occurring at 0.15 mm (see Fig. 1). This highlights that a greater microvasculature perfusion occurs at a deeper subdermal depth in the sacrum [40], which is corroborated by the significantly deeper plexus depth in the sacrum compared to the heel (see Table 1). In contrast, the amount of microvasculature being perfused in the heel is less compared to the sacrum, with peak vascular expression occurring at a more superficial depth. Microvascular morphology is heterogenous due to a number of contributing factors, including age, anatomical location and extrinsic factors [41–43], yet the anatomy of the microcirculation is consistent [40]. The finding provides an important profile of the microvasculature of two of the most affected skin areas by pressure ulcers. It has been widely reported that nearly 30% of pressure ulcers occur at the heel, with this region being the second most likely area for pressure damage after the sacrum [22, 23].

The data shows there were structural anatomical site differences between the sacrum and the heel (as seen in Table 1). Specifically, the OAC and dermal brightness were greater in the sacrum compared to the heel, with high OAC values (>2mm^-1^) in sacrum compared to the lower (<2mm^-1^) values in heel. These data suggest differences in the tissue morphology of the two skin sites, where greater light attenuation occurs due to increased absorption, scattering or both from underlying tissues (potentially due to differences in tissue morphology, e.g., greater collagen content [26, 44]). The surface topography (Rq) was not different between the anatomical sites, but our data are comparable to other skin sites, e.g., the cheek, that have been investigated to determine structural and dynamic skin parameters to quantify skin sensitivity [33]. These data may be applicable not only to clinical applications [26, 45, 46], but also in understanding how tissue integrity is compromised from mechanical shear stress.

Application and use of OCT is both wide and varied [27], however; there is limited available data to quantify its reliability. Our study expanded this knowledge by providing test-retest reliability data at two different anatomical sites in two age cohorts, across three separate visits to our laboratory. Reliability was interpreted using the 95% CI rather than the ICC estimate exclusively, which is considered to provide a more objective range to assess reliability [34]. Table 2 shows agreements for the structural and dynamic parameters and overall absolute agreement ranged between ‘moderate to good’ for both anatomical sites. The exception was epidermal thickness, which is perhaps not surprising given the technical limitations in extracting this parameter. Table 3 shows the absolute agreements for vascular density against depth and demonstrates that overall, our measurements were reliable. D-OCT has been externally validated for the imaging of cutaneous vasculature against laser speckle contrast imaging [47].

Data for the thickness of the epidermis have been included for completeness, however; it is noted that some data sets were included due to limitations in the proprietary OCT Vivotools software. It appears that the A-scans did not have a clear peak at the dermal-epidermal junction, which has been attributed to the digital smoothing of the A-scans [33, 44]. Whilst algorithms may exist to circumvent this issue [33], future research should consider the appropriateness of using Vivotools to analyse epidermal thickness.

The fundamental knowledge on skin properties of sites at risk of PU developed by this study has important applied implications. As noted earlier, several healthcare technologies, including interfaces and support surfaces have been developed to deliver local cooling via microclimate management systems, in an attempt to prevent the loss of skin tissue viability under mechanical loading [12]. However, evidence underlying their efficacy across different populations, and on whether interventions should be adjusted based on factors such as age and skin site, remain limited. The authors recognise some limitations of the study; firstly, the participant cohorts recruited to take part were purposefully screened through self-reporting to be physically active and healthy individuals. Therefore, these data may not be directly applicable to PU risk populations who commonly present with metabolic, cardiovascular and neurological comorbidities that may influence skin integrity. Secondly, temperature and humidity data were not recorded at the local skin sites. Whilst experimentation was conducted in a temperature-controlled laboratory, direct application of our findings to specific cooling interventions may be limited. However, these data form part of a wider investigation that is examining the influence of different levels of cooling on inflammatory, biophysical and perceptual responses to sustained mechanical loading and shearing, at the sacrum [12] and heel [48]. Therefore, our data provide a structural baseline for functional studies investigating localised cooling efficacy, in younger and older healthy individuals.

In conclusion, using non-invasive advanced OCT imaging, our findings indicated that skin epidermal and microvascular properties vary largely between the heel and sacrum, but not because of ageing up to ˜70y. The data presented here provides a unique characterisation of structural and morphological skin parameters that are important for understanding the aetiology of PU formation at these vulnerable skin sites. Importantly, this evidence can help inform design parameters (e.g. cooling magnitude) for the development of healthcare technologies (e.g. support surfaces with microclimate control) that may help to prevent the onset of tissue damage leading to PU.

## Figures and Tables

**Table 1:** Skin epidermal (i.e. OAC, dermal brightness, epidermal thickness, Rq) and microvascular properties (plexus depth) at the sacrum and heel in the younger and older cohort. Data are presented as means and 95% CI and actual sample size available for analysis (N) is reported for each parameter.

	Sacrum		Heel				
Skin parameters	Younger	Older	Younger	Older	Main effect of age (P)	Main effect of anatomical site (P)	Interaction effect (P)
OAC (mm^-1^)	2.8 [2.7-2.9]	2.9 [2.8-3.1]	1.7 [1.4-2.0]	1.7 [1.3-2.0]	0.908	**<0.001**	0.619
***N***	22	17	22	17			
Dermal brightness (%)	93.5 [91.6-95.5]	92.8 [90.6-95.0]	55.0 [51.5-58.4]	54.5 [50.6-58.4]	0.710	**<0.001**	0.199
***N***	22	17	22	17			
Epidermal thickness (μm)	95.6 [83.5-107.7]	92.4 [80.2-104.5]	297.4 [250.8-344.0]	252.6 [206.0-299.1]	0.172	**<0.001**	0.199
***N***	11	11	11	11			
Rq (μm)	20.0 [17.6-22.4]	18.9 [16.2-21.5]	17.7 [15.2-20.2]	17.8 [15.0-20.6]	0.688	0.185	0.646
***N***	22	18	22	18			
Plexus depth (μm)	269.2 [253.3-285.1]	276.5 [259.4-293.6]	125.9 [90.9-160.9]	117.3 [79.6-154.9]	0.961	**<0.001**	0.589
***N***	22	19	22	19			

Abbreviations: OAC, optical attenuation coefficient, Rq, skin surface roughness.

**Table 2:** ICC estimates for structural and dynamic skin properties in the skin of the sacrum and heel on test-rest reliability. ICC estimates and their 95% CI were calculated based on a mean rating (*k* = 3), absolute agreement using a two-way mixed effects model. To evaluate the level of reliability the 95% CI estimates of the ICC were used and interpreted as poor (<0.5), moderate (0.5-0.75), good (0.7-0.9) and excellent reliability (>0.9) [34].

		95% Confidence Interval		F Test with True Value 0				
Sacrum	ICC	Lower Bound	Upper Bound	Value	df1	df2	P	Reliability
OAC (mm^-1^)	0.915	0.856	0.953	11.699	38	76	<0.001	Good to excellent
Dermal brightness (%)	0.767	0.602	0.870	4.208	38	76	<0.001	Moderate to good
Epidermal thickness (μm)	0.605	0.259	0.804	2.512	27	54	0.002	Poor to good
Rq (μm)	0.715	0.518	0.840	3.468	39	78	<0.001	Moderate to good
Plexus depth (pm)	0.828	0.71	0.902	5.689	40	80	<0.001	Good to excellent
**Heel**								
OAC (mm^-1^)	0.903	0.836	0.946	10.169	38	76	<0.001	Good to excellent
Dermal brightness (%)	0.873	0.775	0.930	8.872	38	76	<0.001	Good to excellent
Epidermal thickness (μm)	0.944	0.884	0.976	17.857	19	38	<0.001	Good to excellent
Rq (μm)	0.877	0.792	0.931	8.038	39	78	<0.001	Good to excellent
Plexus depth (μm)	0.752	0.567	0.865	3.97	35	70	<0.001	Moderate to good

Abbreviations: OAC, optical attenuation coefficient, Rq, skin surface roughness.

**Table 3:** ICC estimates of test-rest reliability for vascular density at different depths in the skin of the sacrum and heel, collapsed by age group. ICC estimates and their 95% CI were calculated based on a mean rating (*k* = 3), absolute agreement using a two-way mixed effects model. To evaluate the level of reliability the 95% CI estimates of the ICC were used and interpreted as poor (<0.5), moderate (0.5-0.75), good (0.7-0.9) and excellent reliability (>0.9) [34].

Sacrum							Heel			
		95% Confidence Interval					95% Confidence Interval			
Depth (mm)	ICC	Lower Bound	Upper Bound	P	Reliability	ICC	Lower Bound	Upper Bound	P	Reliability
**0.05**	0.813	0.686	0.894	<0.001	Moderate to good	0.791	0.647	0.882	<0.001	Moderate to good
**0.10**	0.788	0.643	0.880	<0.001	Moderate to good	0.837	0.727	0.908	<0.001	Moderate to good
**0.15**	0.810	0.680	0.893	<0.001	Moderate to good	0.793	0.652	0.883	<0.001	Moderate to good
**0.20**	0.822	0.700	0.899	<0.001	Good	0.801	0.667	0.887	<0.001	Moderate to good
**0.25**	0.790	0.646	0.881	<0.001	Moderate to good	0.792	0.653	0.882	<0.001	Moderate to good
**0.30**	0.731	0.549	0.848	<0.001	Moderate to good	0.784	0.639	0.877	<0.001	Moderate to good
**0.35**	0.662	0.433	0.809	<0.001	Poor to good	0.762	0.603	0.865	<0.001	Moderate to good
**0.40**	0.750	0.579	0.858	<0.001	Moderate to good	0.757	0.593	0.862	<0.001	Moderate to good
**0.45**	0.824	0.704	0.900	<0.001	Good	0.882	0.802	0.933	<0.001	Good to excellent
**0.50**	0.853	0.754	0.916	<0.001	Good to excellent	0.917	0.859	0.953	<0.001	Good to excellent
**0.55**	0.868	0.779	0.925	<0.001	Good to excellent	0.909	0.843	0.949	<0.001	Good to excellent
**0.60**	0.874	0.789	0.928	<0.001	Good to excellent	0.884	0.795	0.936	<0.001	Good to excellent
**0.65**	0.879	0.797	0.931	<0.001	Good to excellent	0.843	0.724	0.913	<0.001	Good to excellent
**0.70**	0.890	0.817	0.938	<0.001	Good to excellent	0.825	0.703	0.902	<0.001	Good to excellent
**0.75**	0.880	0.798	0.932	<0.001	Good to excellent	0.840	0.731	0.909	<0.001	Good to excellent
**0.80**	0.857	0.758	0.919	<0.001	Good to excellent	0.862	0.770	0.922	<0.001	Good to excellent
**0.85**	0.782	0.633	0.877	<0.001	Moderate to good	0.841	0.733	0.910	<0.001	Good to excellent
**0.90**	0.674	0.453	0.815	<0.001	Poor to good	0.771	0.616	0.870	<0.001	Moderate to good
**0.95**	0.417	0.03	0.667	0.019	Poor to moderate	0.711	0.516	0.836	<0.001	Moderate to good
**1.00**	0.296	-0.168	0.598	0.088	Poor to moderate	0.712	0.518	0.836	<0.001	Moderate to good

## Data Availability

All data generated or analysed during this study are included in this article. Further enquiries can be directed to the corresponding author.
